# Delay in diagnosis of pulmonary tuberculosis in low-and middle-income settings: systematic review and meta-analysis

**DOI:** 10.1186/s12890-017-0551-y

**Published:** 2017-12-13

**Authors:** Fentabil Getnet, Meaza Demissie, Nega Assefa, Bizatu Mengistie, Alemayehu Worku

**Affiliations:** 1 Department of Public Health, College of Medicine and Health Sciences, Jigjiga University, PO Box = 1020, Jigjiga, Ethiopia; 2 Addis Continental Institute of Public Health, Addis Ababa, Ethiopia; 3 School of Public Health, College of Health and Medical Sciences, Haramaya University, Harar, Ethiopia; 4 School of Public Health, College of Health Sciences, Addis Ababa University, Addis Ababa, Ethiopia

**Keywords:** Patient delay, Health system, Diagnosis delay, Pulmonary tuberculosis

## Abstract

**Background:**

Assessment of delays in seeking care and diagnosis of tuberculosis is essential to evaluate effectiveness of tuberculosis control programs, and identify programmatic impediments. Thus, this review of studies aimed to examine the extent of patient, health system, and total delays in diagnosis of pulmonary tuberculosis in low- and middle- income countries.

**Methods:**

It was done following the Preferred Reporting Items for Systematic Reviews and Meta-Analyses. Electronic databases were searched to retrieve studies published from 2007 to 2015 including *Pubmed central, Springer link*, *Hinari* and *Google scholar*. Searching terms were *pulmonary tuberculosis, health care seeking, health care seeking behavior, patient delay, diagnostic delay, health system delay, provider delay, and doctor delay.* Retrieved studies were systematically reviewed and summarized using Comprehensive Meta-analysis software.

**Results:**

Forty studies involving 18,975 patients qualified for systematic review, and 14 of them qualified for meta-analysis. The median diagnostic delay ranged from 30 to 366.5 days [IQR = 44–77.8], with a 4–199 days [IQR = 15–50] and 2–128.5 days [IQR = 12–34] due to patient and health system delays, respectively. The meta-analysis showed 42% of pulmonary tuberculosis patients delayed seeking care by a month or more; uneducated patients [pooled OR = 1.5, 95%CI = 1.1–1.9] and those who sought initial care from informal providers [pooled OR = 3, 95%CI = 2.3–3.9] had higher odds of patient delay.

**Conclusion:**

Delay in diagnosis is still a major challenge of tuberculosis control and prevention programs in low- and middle- income settings. Efforts to develop new strategies for better case-finding using the existing systems and improving patients’ care seeking behavior need to be intensified.

## Background

Tuberculosis (TB) causes an estimated 10.4 million cases and 1.7 million deaths globally, with the heaviest toll in Low- and Middle-Income countries (LMICs) [1]. The main strategies to control TB are early diagnosis and prompt treatment initiation [2]. Passive case-finding is the main approach currently applied by most national TB control programs (NTPs) [3]. The term “passive” implies that TB case detection entirely relies on people with TB symptoms presenting themselves to the health facilities [2]. Successful case detection also relies on health systems capacity to promptly diagnose and commence treatment [4].

However, this passive process has not been as effective as it should be in many LMICs [5]. The effectiveness of the passive case-finding system is influenced by many factors including patient health seeking behavior, the efficiency and competence of healthcare workers, and the quality of laboratory facilities [6, 7]. Failure to timely detect and treat TB could worsen illness severity, prolongs patient suffering, increases the risk of patient death, and facilitates the transmission of the disease (if smear positive pulmonary TB) to close contacts [8–10].

Delay in diagnosis and treatment initiation of TB has remained unacceptably high especially among high burden countries [6, 7]. Related systematic reviews were reported on the subject matter in 2008 [6] and 2010 particularly from Sub-Saharan Africa [11]. These reviews included all studies done on delay in diagnosis and treatment of TB regardless of the disease type. However, most NTPs promote healthcare seeking for presumptive cases with pulmonary symptoms [12]. Plus, pulmonary tuberculosis (PTB) patients are responsible for disease transmission (specially smear positive patients), and longer delays facilitate the spreading to close contacts [13]. On the other side, extra-pulmonary TB (EPTB) forms are not easily detectable using clinical and laboratory examinations. As a result, delay will inflate if EPTB patients are included [2].

More importantly, massive efforts were implemented during the era of Stop TB Strategy (2006–2015) to substantially reduce the global burden of TB by 2015 through universal access to diagnosis and treatment regardless of socio-economic barriers [14]. A periodic conduct of systematic review is warranted to incorporate lessons learned from such massive and dynamic TB control programs to improve the practice of early detection and initiation of treatment. Therefore, this systematic review and meta-analysis was conducted to summarize the extent of delays and identify factors that influenced prompt detection and treatment of PTB in LMICs where access or utilization of TB services has not been optimal as required. We have also examined the median delay differences between Sub-Saharan Africa and Other LMICs.

## Methods

We systematically reviewed studies reported from LMICs to summarize delays in diagnosis of PTB patients and to identify factors contributing to high extents of delay. We thought such a specific study on delay in diagnosis of PTB patients would be more beneficial from programmatic and interventional perspectives. Meta-analysis principles were applied to combine numeric data on delays and affecting factors. We followed the Preferred Reporting Items for Systematic Reviews and Meta-Analyses (PRISMA) [15] as a standard guideline.

### Search strategy

Various electronic databases were searched to retrieve relevant published studies using Boolean searching technique [16]. We used ‘AND’ and ‘OR’to connect key search terms to retrieve studies from *Pubmed*. The search terms we used include *pulmonary tuberculosis, diagnosis delay, patient delay, health system delay, provider delay, doctor delay, delayed consultation, health care seeking, and health care seeking behavior* (Table 1). In addition, we searched other web-based sources including *Springer link*, *Hinari,* and *Google scholar,* though not standard sources, to retrieve studies that are not indexed in *Pubmed.*

**Table 1:** Pudmed search strategies

S. No.	Search terms or strategies	Remarks
1.	Pulmonary tuberculosis AND diagnosis Delay*	**Example of search detail (1)**: *(“tuberculosis, pulmonary”[MeSH Terms] OR (“tuberculosis”[All Fields] AND “pulmonary”[All Fields]) OR “pulmonary tuberculosis”[All Fields] OR (“pulmonary”[All Fields] AND “tuberculosis”[All Fields])) AND ((“diagnosis”[Subheading] OR “diagnosis”[All Fields] OR “diagnosis”[MeSH Terms]) AND delay[All Fields])* *indicates truncation
2.	Pulmonary tuberculosis AND delayed consultation*	
3.	Pulmonary tuberculosis AND patient delay*	
4.	Pulmonary tuberculosis AND healthcare seeking	
5.	Pulmonary tuberculosis AND healthcare seeking behavior	
6.	Pulmonary tuberculosis AND health system delay*	
7.	Pulmonary tuberculosis AND provider delay*	
8.	Pulmonary Tuberculosis AND doctor delay*	
9.	Patient delay OR health system delay*	
10.	Patient delay OR provider delay*	
11	Patient delay OR doctor delay*	
12.	Tuberculosis AND Diagnosis delay OR delayed consultation OR patient delay OR health system delay OR provider delay OR doctor delay	

We searched each source twice. The first author (FG) conducted the primary searching of studies from sources listed above and then the co-author (NA) independently and blindly conducted it again using similar searching technique, databases and terms. All the potential studies identified by both authors were set for screening and selection process. We contacted 5 authors via email for unpublished studies and raw data but none responded to our requests.

### Inclusion/exclusion criteria

We included studies that were reported in English, conducted in low and middle income countries, published between 2007 and 2015 (era of Stop TB strategy), with any observational study design (cross-sectional, case-control, or cohort studies), and those which fully or partially measured diagnosis delays (patient delay/health system delay/total diagnosis delay). With respect to the study participants, we included studies on pulmonary TB patients who sought healthcare themselves and above 15 years old regardless of smear type (positive/negative/unknown) and treatment category (new/retreatment). Studies done on PTB and EPTB patients were considered when data were presented for PTB patients separately. However, we excluded studies that were on presumptive TB cases (formerly called ‘*suspects’*) and special groups (e.g. HIV, MDR), qualitative in design, and community based studies which applied active case-finding strategies.

Further inclusion criteria were used for Meta-analysis from eligible studies for systematic review. These included studies that categorized patient delay using 15, 21 or 30 days as cut-off points and reported cross tabulations and/or odds ratio/relative risk of explanatory variables.

### Selection of studies

Eligible studies were selected using the pre-specified inclusion/exclusion criteria. Initially, the studies were searched and potential articles were identified using the title and then abstract by two reviewers (FG and NA) independently. Following identification of potential studies, FG made the final selection through thorough review of full articles including the study design, participants, outcome variable studied (delay), and publication year. Co-authors (NA and BM) closely supervised the selection process.

### Quality assessment

All included studies were assessed for their scientific quality usingquality assessment tools developed by the National Collaborating Center for Environmental Health [17] and National Institute of Health (NIH) National Heart, Lung and Blood Institute [18].

The quality of each study was assessed using 4 parameters: 1) defined study population, 2) representativeness of participants and low non-response rate, 3) comparability of analysis groups and outcome ascertainment, and 4) adjusted analysis to control confounding. Each parameter was rated as 1 if ‘Yes’ and 0 if ‘No’. Then the scores were added and the quality of each studywas leveled as good (if 3 or 4 ‘yes’s), fair (2 ‘yes’s) and poor (if 0 or 1 yes). Fair and good quality studies were considered as satisfying quality (Table 2). The quality of all included studies was assessed by first author (FG), followed by immediate and independent crosschecking by two co-authors (NA and BM). Discrepancies were solved through mutual understanding.

**Table 2:** quality assessment result of the studies included in the systematic review and meta-analysis

Study	Study Design	A	B	C	D	Total score
Alavi et al. 2015 [45]	CS	1	0	1	1	3
Asefa et al. 2014 [27]	CS	1	1	1	1	4
Aye et al. 2010 [46]	RC	1	0	1	1	4
Basnet et al. 2009 [47]	CS	1	1	1	1	4
Behera et al. 2013 [48]	CS	1	1	1	1	4
Belkina et al. 2014 [44]	CS	1	1	1	1	4
Biya et al. 2010 [49]	CS	1	0	1	1	3
Buregyeya et al. 2014 [50]	CS	1	0	1	1	3
Cambanis et al 2007 [51]	CS	1	1	1	1	4
Deponti et al. 2013 [52]	CS	1	0	1	1	3
Ekinci et al. 2014 [22]	CS	1	0	1	1	3
Ford et al. 2009 [21]	CS	1	0	1	1	3
Gebeyehu et al. 2014 [34]	CS	1	0	1	1	3
Gosoniu et al. 2008 [19]	CS	1	0	1	1	3
Gosoniu et al. 2008 [19]	CS	1	0	1	1	3
Gosoniu et al. 2008 [19]	CS	1	0	1	1	3
Huong et al. 2007 [30]	CS	1	1	1	1	4
Hussen et al. 2012 [35]	CS	1	0	1	1	3
Jurcev-Savicevic et al. 2013 [53]	CS	1	1	1	1	4
Kansiime et al. 2013 [54]	CS	1	1	1	1	4
Li et al. 2012 [55]	CS	1	1	1	1	4
Maamari et al. 2008 [25]	CS	1	1	1	1	4
Maciel et al. 2010 [56]	CS	1	1	1	1	4
Maior et al. 2012 [57]	CS	1	1	1	1	4
Makwakwa et al. 2014 [58]	CS	1	1	1	1	4
Mesfin et al. 2009 [29]	CS	1	1	1	1	4
Mfinanga et al. 2008 [59]	CS	1	1	1	1	4
Mohamed et al. 2013 [36]	CS	1	1	1	1	4
Nasehi et al. 2012 [60]	CS	1	1	1	1	4
Ngadaya et al. 2009 [61]	CS	1	1	1	1	4
Ngangro et al. 2012 [62]	CS	1	1	1	1	4
Pradhan et al. 2010 [43]	CS	1	1	1	1	4
Rabin et al. 2013 [63]	CS	1	1	1	1	4
Sabawoon et al. 2011 [31]	CS	1	0	1	1	3
Saifodine et al. 2013 [37]	CS	1	1	1	1	4
Sendagire et al. 2010 [64]	CS	1	1	1	1	4
Takarinda et al. 2015 [32]	CS	1	1	1	1	4
Tamhane et al. 2012 [23]	CS	1	0	1	1	3
Ukwaja et al. 2013 [24]	CS	1	1	1	1	4
Woith et al. 2014 [65]	CS	1	0	1	1	3
Yimer et al. 2015 [66]	CS	1	1	1	1	4

CS; refers to cross-sectional and RC; refers to retrospective cohort. The columns: A. Study population defined; B. representativeness of participants and low non-response rate; C. Comparability of analysis groups and outcome ascertainment; D. analysis to control confounding

Rating: (0 = No and 1 = Yes). This format was adapted from a similar review done in China [67]

### Data extraction

Excel spreadsheet was used to extract available data on the name of first author, country where the study was conducted, publication year, type of health-care facility (study setting), study design, sample size, and median and/or mean delays (patient, health system and total) in days. Data from a study by Gosoniu et al. that involved 3 countries (Bangladesh, India and Malawi) was extracted separately for each country [19]. Delays reported in weeks were transformed into days by a multiple of 7 (Tables 3 and 4). The predictor variables that have statistically significant association with patient, health system and total diagnosis delay were also extracted (Table 5).

**Table 3:** The median delay (days) in diagnosis of pulmonary tuberculosis in Sub-Saharan Africa, 2007 to 2015 (*n* = 19)

First Author	Country	Publication Year	Design	Setting	Sample Size	Patient Delay	Health System Delay	Total Delay	Ref. No
Cambanis	Cameroon	2007	CS	Hospital	243	15	*	*	[51]
Mfinanga	Tanzania	2008	CS	Hospital, HC& dispensaries	639	14 (Ẋ =37.4)	*	*	[59]
Gosoniu	Malawi	2008	CS	Health centers, clinics	100	*	*	33.5	[19]
Mesfin	Ethiopia	2009	CS	Hospital & HC	924	30	*	*	[29]
Ngadaya	Tanzania	2009	CS	Hospital, HC,TB dispensary	206	62	*	90 (Ẋ =125.5)	[61]
Biya	Nigeria	2010	CS	Hospital	160	30	*	*	[49]
Sendagire	Uganda	2010	CS	Public health facility	253	28	28	56	[64]
Hussen	Ethiopia	2012	CS	Hospital & HC	129	63	34	97	[35]
Ngangro	Chad	2012	CS	Hospital	286	15	36	57.5	[62]
Ukwaja	Nigeria	2013	CS	Hospital	450	56	21	77	[24]
Kansiime	Uganda	2013	CS	Referral hospital	266	*	9	*	[54]
Saifodine	Mozambique	2013	CS	Hospital and HC	622	61	62	150	[37]
Mohamed	Sudan	2013	CS	TB management unit	292	4 (Ẋ = 27.2)	*	*	[36]
Makwakwa	Malawi	2014	CS	Hospital	588	14	59	80	[58]
Gebeyehu	Ethiopia	2014	CS	Hospital &HC	153	28	*	*	[34]
Asefa	Ethiopia	2014	CS	Health center	328	30	7	37	[27]
Buregyeya	Uganda	2014	CS	Hospital and HC	158	30	77	112	[50]
Yimer	Ethiopia	2015	CS	Hospital &HC	231	21	*	*	[66]
Takarinda	Zimbabwe	2015	CS	Hospitals, clinics, PHF	383	28	2	30	[32]

Key: * indicates data not available

**Table 4:** The median delay (days) in diagnosis of pulmonary tuberculosis in low and middle income countries other than Sub-Saharan Africa, 2007 to 2015 (*n* = 22)

First Author	Country	Publication Year	Design	Setting	Sample size	Patient Delay	Health System Delay	Total Delay	Ref. No
Huong	Vietnam	2007	CS	TB units	2087	21	7	30	[30]
Maamari	Syria	2008	CS	TB centers	800	31 (Ẋ = 52.7)	11.7 (Ẋ = 24.8)	55 (Ẋ = 77.6)	[25]
Gosoniu	Bangladesh	2008	CS	Clinics, HC	102	*	*	60	[19]
Gosoniu	India	2008	CS	Health centers, Clinics	127	*	*	74	[19]
Basnet	Nepal	2009	CS	Hospital &HC	307	50	18	60	[47]
Ford	Peru	2009	CS	Hospital & Health post	108	61	*	*	[21]
Maciel	Brazil	2010	CS	Primary PHF	304	76	30	110	[56]
Aye	Tajikistan	2010	RC	Hospital, HC, clinics	204	21.5	16	53	[46]
Pradhan	India	2010	CS	DOTs centers	266	6 (Ẋ = 30)	31 (Ẋ = 61)	37 (Ẋ = 91)	[43]
Sabawoon	Afghanistan	2011	CS	Hospital & clinic	122	199 (Ẋ = 205.2)	128.5 (Ẋ = 150)	366.5 (Ẋ = 356)	[31]
Li	China	2012	CS	TB clinics	323	10 (Ẋ =31)	*	*	[55]
Nasehi	Iran	2012	CS	Hospital, clinics	5702	*	*	59	[60]
Tamhane	India	2012	CS	Health center	150	15	31	*	[23]
Behera	India	2013	CS	Tertiary hospital	204	16 (Ẋ =32.97)	39 (Ẋ = 60.46)	43 (Ẋ = 75.7)	[48]
Rabin	Georgia	2013	CS	TB diagnostic centers	247	23.5 (Ẋ = 56.2)	14(Ẋ = 33.7)	59.5 (Ẋ = 89)	[63]
Deponti	Brazil	2013	CS	Tertiary hospital	153	30	18	60	[52]
Jurcev-Savicevic	Croatia	2013	CS	Hospital	241	*	15	*	[53]
Woith	Russia	2014	CS	TB clinics	105	30	*	*	[65]
Ekinci	Turkey	2014	CS	Referral hospital	136	12 (Ẋ = 30.5)	29 (Ẋ = 48.7)	54 (Ẋ = 82.2)	[22]
Alavi	Iran	2015	CS	Health Center	139	*	*	48 (Ẋ = 73)	[45]
Belkina	Uzbekistan	2014	CS	Hospitall	538	27	7	50	[44]
Maior	Brazil	2012	CS	TB clinic	199	56	14	70	[57]

Key: * Value not obtainable

Ẋ: Mean value

CS: Cross-sectional

HC: Health center

PHF: Public health facility

RC: Retrospective cohort

**Table 5:** Summary of risk factors for delay in diagnosis in low and middle income countries, 2007 to 2015

Patient delay	Health System Delay	Total Delay
Socio-demographic: Poor literacy [21, 24, 28, 29, 35, 62], Rural residence [29, 34, 35], Urban residence [24, 36, 37], Older age [24], long distance to the nearest HF [24, 30–32, 35, 59], Being female [30, 32, 36, 59, 63], Being male [21, 50] Socio-economic: Average monthly working days > 24 [55], Unemployment [36, 59], Being main income earner [51], Low economic status [36, 61]	Socio-demographic: Older age [24, 60], Long distance to nearest HF [24, 25, 44], Being female [19, 60, 63, 68], Male sex [24, 27, 48, 50] Socio-economic: Low income [43], Unemployment [44], Labor migration [44]	Socio-demographic: Being female [19, 60, 63, 68],Being male [24, 27, 48, 50],Being housewife [19], Higher family size [27], Poor literacy [27],Urban residence [24, 60],Long distance to nearest HF [24, 25, 44], Older age [24, 60], General caste [48]
Behavioral: Evil/bad luck perception [29], Knowledge of coughing > 3 wks as PTB symptom [58], Poor knowledge of PTB symptoms [25, 37, 48, 49, 57, 59, 61], Perceived social stigma [31, 51], Perceived inability to pay for care [23], Perception that PTB is common [21, 36], Belief of PTB is associated with HIV [59, 61, 64], Perceiving symptoms as non-serious [22, 43], Belief of PTB is curable [21, 64]	Clinical: Good functional status [35], No cough [56], Unusual symptoms other than common ones [53], Normal chest X-ray [56], Presence of fever [54], Fibrotic changes on chest x-ray ® [52], Smear negativity [28, 37, 53, 54, 58]	Behavioral: Perceived stigma [25, 27], Sanitation as perceived cause [19],1st visit to informal provider (traditional, religious [24, 25], Self-medication [44], Smoking [45]
Initial Care seeking: 1st visit at informal provider (traditional, religious) [25, 29, 31, 35, 37, 58], Self-medication [31, 32, 44, 51, 52, 62, 63], Alcoholism [44], smoking /tobacco use [47, 63]	Initial Care seeking: 1st visit to private facilities (clinics, drug vendors) [29–31, 35, 44, 50, 58], 1st visit to informal provider (traditional, religious) [23, 24], 1st visit to lower level HF [30, 32, 46, 49], Contact with more than two health providers [35]	Initial Care seeking: Prior antibiotics medication, 1st visit to private facility [44, 50, 60], Multi-facility visit [25]
Clinical: Smear positivity [29, 47], HIV positivity [44], Being new case [58], Chronic/persistent cough [44, 56, 63], Pulmonary co morbidity [37, 63], Mild severity of illness [35], MTB lineage 7 [66], Hemoptysis® [55, 62, 63], Chest pain® [61], Multiple symptoms® [63]		Clinical: Chronic cough [19, 44, 63], Only chest pain [19], Immunosuppressive therapy (62)

Key: ®: representsthe association is negative (preventive)

*HF* Health facility, *MTB Mycobacterium tuberculosis*

Further data were extracted on the event (delay) among exposure categories and/or odds ratio for meta-analysis. Health system and total delays were not considered for meta-analysis because of lack of clear cutoff values. The studies we reviewed did not use standard cutoff value to categorize patient delays. Therefore, we used the WHO recommended cutoff points (15 and 21 days) [20] and the commonly used 30 days by numerous studies. There was also considerable variability in the way studies categorized explanatory variables, thus variables categorized in the same way were considered for meta-analysis. The final explanatory variables we came up with, therefore, were sex, residence, educational status, first care seeking from informal healthcare provider, and HIV status.

### Definition of terms

The definitions of terms in included studies were critically reviewed. Different studies used different terms to describe patient, health system, and diagnosis delays. For instance, some studies described patient delay as TB test delay or TB test seeking delay [21] and patients’ application interval [22]. Health system delay is also referred to as doctor delay or provider delay [22–24]. Most studies used the term ‘total delay’ as the time until the diagnosis while some until treatment. In such a case, we took the delay until diagnosis [25–29]. Accordingly, delay terms have been defined as follows:

### Patient delay

The time interval between the onsets of patient recognized PTB symptom(s) recognize and the patient’s first consultation to a healthcare provider.

### Health system delay

The time interval between the patient’s first consultation with a health care provider and the date of diagnosis.

### Diagnosis/Total delay

**Fig. 1:**
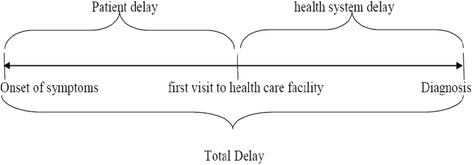
Illustration of patient, health system and total delay

The time interval between the onset of PTB symptom(s) and the date of diagnosis. Alternatively, the sum of patient delay and health system delay (Fig. 1).

### Data analysis

#### Systematic review

The data organized on excel spreadsheet were exported to SPSS version 21 for analysis. The median delay (in days) was summarized using median, box plots, inter quartile ranges (25th and 75th percentile), and 95% confidence interval and ranges. Since the distribution of median delays was skewed, the pooled median delay was taken rather than the mean to compare delays between Sub-Saharan Africa and other LMICs. The median differences were estimated using the non-parametric Mann-Whitney test which was used to test the statistical difference of pooled median delays between Sub-Saharan Africa and other LMICs. In addition, the 95% confidence interval was calculated using bootstrap method.

#### Meta-analysis

The data for each of the six predictor variables were entered into the Comprehensive Meta-analysis software version 2. The effect size measurement computed were odds ratio (pooled and individual) and prevalence of patient delay at 30 days cutoff point. Forest plots were drawn to visualize effect size (odds ratio with 95%CI). Both fixed and random effect models were used for pooled analysis based on the heterogeneity level of studies included. Heterogeneity was evaluated using Cochrane Q and I^2^ tests as well as Q/df (degree of freedom) ratio. Cochrane Q test (*p* = 0.1), Q/df = 1and I^2^ = 50% were considered as cutoff points to mark heterogeneity and to select the effect model for combined analysis.

As initial test of pooled analysis, we used fixed effect model to combine individual effect sizes. If there was no heterogeneity observed during initial test using fixed effect model (i.e. *P* > 0.1 and I^2^ ≤ 50% and Q/df ≤ 1**or** *p* ≤ 0.1 but I^2^ ≤ 50%), we used fixed model as final model to estimate combined effect sizes. However, if there was significant heterogeneity observed (i.e. p ≤ 0.1 and I^2^ > 50%, **or** *p* > 0.1 but Q/df > 1), we used random effect model. The sources of heterogeneity were assessed by subgroup analysis based on clinical heterogeneity of the studies mainly using smear status of participants. Funnel plot and Egger’s regression test were used for checking graphic and statistical publication biases, respectively. Sensitivity test was done to check the effect of each study on combined effect size.

## Results

### Description of selected studies

**Fig. 2:**
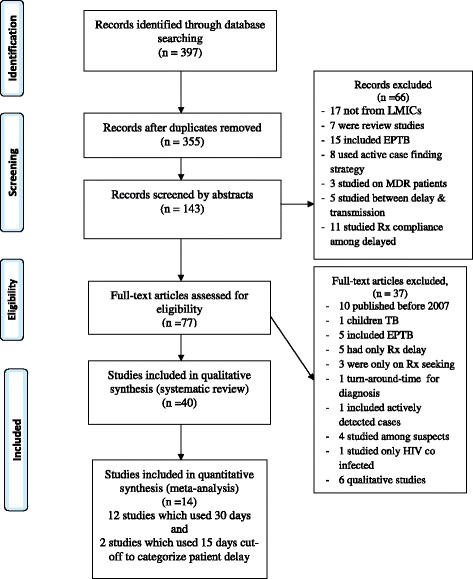
PRISMA flow diagram for article selection and screening

A total of 40 eligible studies were retrieved for systematic review; 19 (47.5%) from sub-Saharan Africa, 20 (50%) from other LMICs, and 1 (2.5%) study involved three countries (Bangladesh, India and Malawi). Among these, 14 studies were found to be eligible for meta-analysis, two with 15 days and 12 with 30 days cutoff points to categorize patient delay (Fig. 2). The numbers of studies which measured patient, health system and total median delays were 35, 27 and 30 respectively (Tables 3 and 4). All but one study used cross-sectional study design with either one time or prospective participant recruitment strategy.

There was certain level of variation on categorization of delays across reviewed studies. Majority of the studies categorized time in diagnosis into ‘delayed’ and ‘not delayed’ using cutoff point. However, there was no uniform cutoff point across all studies. Some studies used the median of their finding, and others used 2 weeks (15 days), 3 weeks (21 days), 1 month (30 days). With no justification, one study used 6 and 12 weeks as cutoff point to determine patient and total delays, respectively [30]. A few studies considered delay as continuous variable, and computed linear regression, ANOVA and Mann-Whitney test.

### Diagnosis delay overview

The overall median delay in LMICs ranged from 4 to 199 days (25th & 75th percentile = 15 & 50) for patient delay, 2 to 128.5 days (25th & 75th percentile = 12 & 34) for health system delay, and 30 to 366.5 days (25th& 75th percentile = 44 & 78.8) for total delay. All of the highest median patient (199), health system (128.5) and total (366.5) delays were reported from Afghanistan [31]. The mean patient delay (35.5 days, 95%CI = 24.4–55.4) was similar to mean health system delay (28.7 days, 95%CI = 19.7–45.8), *p* > 0.05.

### Patient delay

**Fig. 3:**
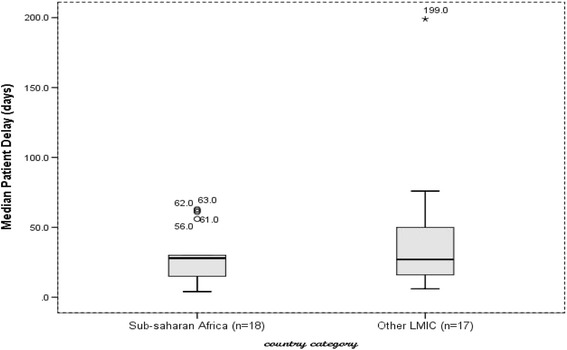
median patient delay in Sub-Saharan Africa and other Low- and Middle-Income countries, 2007 to 2015

The median patient delay in Sub-Saharan Africa varied from 4 days in Sudan to 63 days in Ethiopia, and median of the reported median delays was 28 days (95%CI = 18.9–41.7). In other LMICs, it varied from 6 in India to 199 days in Afghanistan, and median of the reported median delays was 27 days (95%CI = 16.5–55.4). There was no statistically significant difference observed between the two groups, *p* = 0.49 (Fig. 3).

Our review identified a wide range of factors affecting patient delay that were related to individual, health system and clinical characteristics. Some of the individual factorswere poor literacy, first care seeking from informal providers, self-medication, sex (mostly female), wrong perceptions and rural residence among others. The clinical factors were being a new case, mild severity of illness, chronic/persistent cough and pulmonary co-morbidity. On the contrary, the presences of hemoptysis, chest pain and multiple symptoms reduced patient delay (Table 5).

### Health system delay

**Fig. 4:**
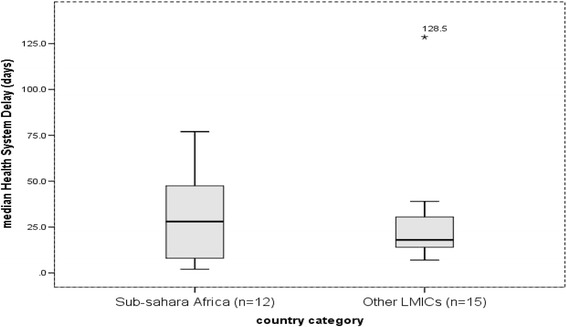
median health system delay in Sub-Saharan Africa and other Low- and Middle-Income countries, 2007 to 2015

The median health system delay in Sub-Saharan countries ranged from the lowest of 2 days in Zimbabwe to 77 days in Uganda and the median of reported median delays was 28 days (95%CI: 14–35.9). In other LMICs, it varied from 7 days in Vietnam and Uzbekistan to 128.5 days in Afghanistan, and the median of reported median delays was 18 days (95%CI: 14–30). However, no median difference between the two groups was observed, *p* = 0.35 (Fig. 4).

Individual factors related to health systems delay were poor literacy, sex (mainly being female), being unemployed and labor migrant while good functional status, absence of cough, presence of unusual symptoms, normal chest X-ray, and smear negativity results were among the clinical factors. First care seeking at private or lower-level health facility was also identified as determinant of health system delay (Table 5).

### Total delay

**Fig. 5:**
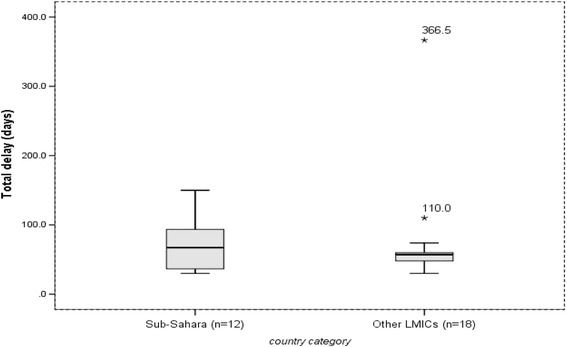
median total delay in Sub-Saharan Africa and other Low- and Middle-Income countries, 2007 to 2015

The median total delay in Sub-Saharan countries ranged from the lowest of 30 days in Zimbabwe to 150 days in Mozambique, and the median of reported median total delays was 67 days (95%CI: 37–90). In other LMICs, it varied from the lowest median delay of 30 days in Vietnam to 366.5 days in Afghanistan, and the median of reported median total delays was 57 days (95%CI: 49–66.8). There was no median difference between the two groups, *p* = 0.31. Outlier median total delays of 110 and 366.5 were reported from Brazil and Afghanistan, respectively (Fig. 5). The determinants of total delay were a mix of factors affecting patient or health system delay (Table 5).

### Summary of findings from meta-analysis

**Fig. 6:**
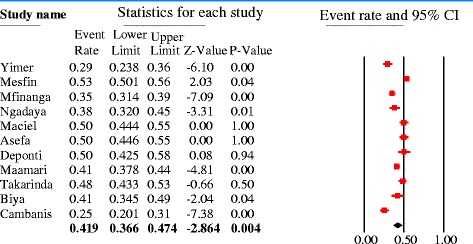
Forest plot showing the pooled proportion of patient delayed above 30 days to seek formal medical care

The combined analysis of 12 studies indicated that 42% (95%CI = 37–47%) of PTB patients delayed a month or more without seeking formal medical care (Q = 123, *p* < 0.1, df = 10, I^2^ = 91%). Significant heterogeneity existed following subgroup analysis by smear type (any type and smear positive) (Fig. 6).

Pooled effect sizes were estimated to determine the independent effect of sex, residence, educational status, first care seeking from informal provider and HIV status on patient delay, and 11, 4, 9, 4 and 3 studies (with 30 days cutoff point) were analyzed, respectively. In addition, 2 studies (with 15 days cutoff) were also pooled.

**Fig. 7:**
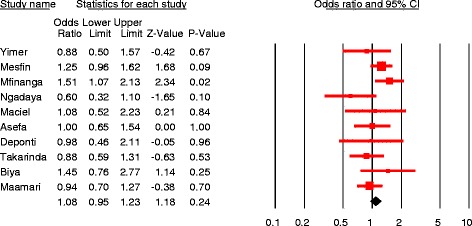
Forest plot showing the association between sex and patient delay at 30 days cutoff

**Fig. 8:**
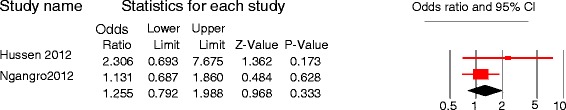
Forest plot showing the association between sex and patient delay at 15 days cutoff

**Fig. 9:**
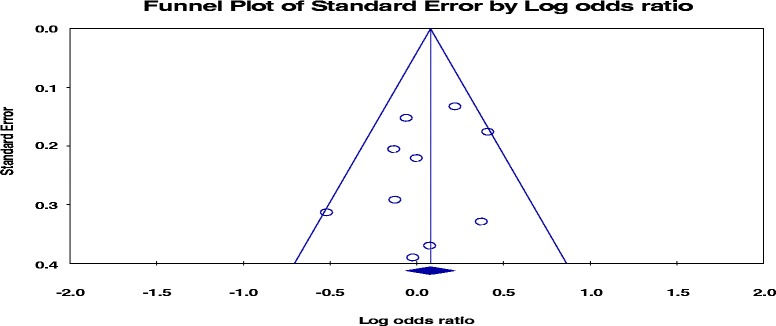
funnel plot of the standard error of pooled proportion of patient delaye above  30 days

**Fig. 10:**
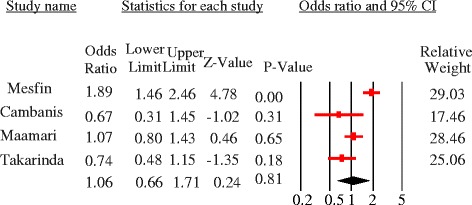
Forest plot showing the association between residence and patient delay at 30 days cutoffs

**Fig. 11:**
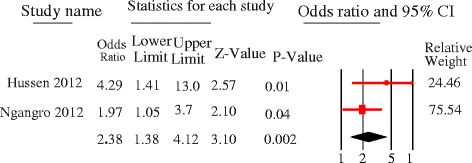
Forest plot showing the association between residence and patient delay at 15 days cutoffs

The majority of studies identified females were more likely to delay but the quantitative summary indicated no statistically significant difference in patient delay between female and male PTB patients at 30 days [pooled OR = 1.08, 95%CI = 0.95–1.23, *p* = 0.24] (Fig. 7) and 15 days cutoff points [pooled OR = 1.26, 95%CI = 0.79–1.99] (Fig. 8). No heterogeneity was observed at both cutoff points (Fig. 9). Similarly, there was no statistically significant difference in patient delay between rural and urban PTB patients at 30 days cutoff [pooled OR = 1.06, 95%CI = 0.66–1.71, *p* = 0.81] (Fig. 10) but observed at 15 days cutoff [pooled OR = 2.4, 95%CI = 1.4–4.1, *p* = 0.002] (Fig. 11).

**Fig. 12:**
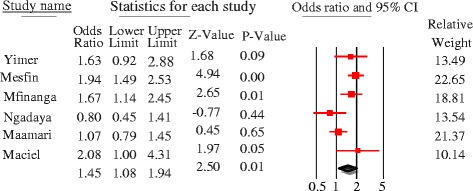
Forest plot showing the association between educational status and patient delay at 30 days cutoff

**Fig. 13:**
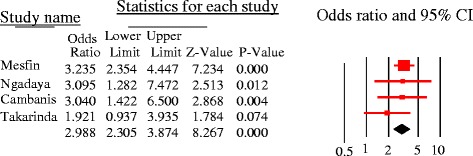
forest plot showing the association between 1st care seeking from informal provider and patient delay of PTB patients (30 days cutoff)

**Fig. 14:**
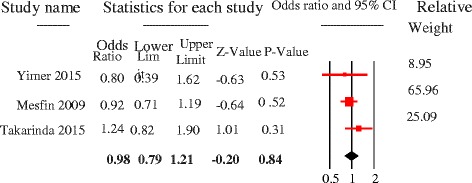
Forest plot showing the association between HIV status and patient delay at 30 days cutoff

Literacy had significant association [pooled OR =1.5, 95%CI = 1.1–1.9, *p* = 0.01], and the odds of patient delay was greater among illiterate PTB patients as compared to those who had formal educition. Significant heterogeneity was observed [Q = 14.3, p = 0.01, df = 5, I^2^ = 65%] (Fig. 12). First care seeking from informal providers had statistically significant association with patient delay at 30 days cutoff [pooled OR = 3, 95%CI = 2.3–3.9, *p* < 0.05]; PTB patients who sought first care at informal providers were 3 times more to delay by a month or more to seek care compared to their counterparts. No heterogeneity was observed [Q = 1.7, *p* = 0.64, df = 3, I^2^ = 0%] and Egger’s regression test, *p* = 0.23 (Fig. 13). The patient delay had no statistically significant association with HIV [pooled OR = 0.98, 95%CI = 0.79–1.21, *p* = 0.84] (Fig. 14).

## Discussion

Our systematic review and meta-analysis indicated that the extent of delay in diagnosis of PTB remains a serious challenge for TB control programs in low- and middle- income countries. Half of the studies in this review reported a median diagnosis delay higher than 2 months, both patient and health system delays made comparable contributions.

This review shows the extent of delays in diagnosis of PTB continued to be as bad as it was a decade ago [6]. Our review showed no significant difference in patient, health system and total delays of PTB cases between Sub-Saharan Africa and other LMICs. Delays have been a common problem to all LMICs which harbor a large share of global TB burden. The minimum median total delay in diagnosis was 30 days in this review [30, 32] which has been indicated as a turning point at which a significant increase in risk for TB transmission occurs [33]. WHO recommends diagnosis within 2 to 3 weeks of symptom initiation [20] though the acceptable time delay is not known. Considering the WHO cutoff point recommendations, this review has, therefore, indicated unacceptable level of delay occurring in LMICs, and actual delays may even be worse than reported here as patients are likely to recall the date when the illness gets severe but not exact date of illness onset.

In this review, the qualitative and quantitative summary of studies indicated the observed patient, and health system delays in LMICs were attributed by a set of individual and health system related factors. The individual factors are related to characteristics of the patients and skill of healthcare providers that influence the health seeking behavior of patients, and suspicion and detection of the disease by healthcare providers. These include demographic, socio-economic, knowledge, beliefs, perceived barriers, behaviors, clinical features and provider skills (*the details stated below*). The health system factors comprise factors which drive within the health system, and hinder access to health facilities and limit availability of diagnosis services. These include limited access of health facilities near to villages, lack of TB diagnosis services in easily accessible health facilities such as private facilities, drug vendors and lower level facilities (e.g. health posts), high cost of some diagnostics (pathology and X-ray), and existence of informal healers (traditional and religious) providing unlimited therapeutic services.

Even though higher number of studies (five) reported that female patients were more likely to wait longer periods without medical consultation, the pooled analysis indicated that sex was not the independent determinant of patient delay (*p* = 0.24). This may be due to gender based social influence in health decision making that might vary across cultures and countries. Similarly, our pooled analysis showed residence was not the independent determinant of patient delay, but three studies in Ethiopia [29, 34, 35] reported rural residence and three other studies from Iran, Nigeria and Sudan [24, 36, 37] reported urban residence as independent determinants of patient delay. This may be owing to the health system of the respective countries. For instance, most patients in rural areas of Ethiopia have primary access to the health post (first level of the health system) where there are no TB diagnostic services [38] and patients might have to walk for some hours to access hospitals or health centers. This is supported by considerable number of studies in our review that indicated patients who travel long distance to access healthcare services experienced longer patient delays in both settings.

Our pooled analysis showed that illiterate patients were more likely to delay in seeking care compared to those patients who had formal education (pooled OR = 1.5; 95% CI = 1.1–1.9). Illiteracy may limit patients’ access to written health education and communication materials that are commonly utilized to increase public awareness about the disease [5]. Our review also found poor knowledge of PTB sign and symptoms, cause and treatment lengthened patient delay in LMICs. Patients who believed the cause of TB is evil/bad luck, perceived the symptoms are common and not dangerous, feared social stigmatization/embarrassment, and associated PTB with HIV sought medical consultation after long suffer from the disease. This is because such limited knowledge and perceived barriers direct patients to hide their illness, self-medicate and practice traditional healing [39]. In addition, poor understanding of the disease in the community results in being discriminated by peers, friends, neighbors and so on [40].

Our meta-analysis identified first care seeking from informal providers as a key determinant of patient delay (pooled OR = 3; 95%CI = 2.3–3.9). Similarly, another review identified patients consulting traditional healers first have experienced patient delay usually in rural residents of Sub-Saharan Africa [11]. This is because patients who have poor knowledge of PTB cause and/or believe PTB is acquired from evil curse deemed trust on treatment by traditional healers or religious leaders to be freed from evil spirits [41]. This implies traditional/religious healers are still preferred destinations for substantial number TB patients. Therefore, engaging them could help to identify presumptive cases early, establish referral link to TB care centers, avoid traditional therapy, and to target community misperceptions and wrong beliefs that hassled healthcare seeking.

Patients who had chronic cough, pulmonary co-morbidity and mild severity of illness delayed seeking care. This may be because patients with such type of symptoms may suspect other common respiratory syndromes that are not considered serious. However, the presence of hemoptysis, chest pain and multiple symptoms reduced the possibility of patient delay. Beyond delaying care seeking, clinical presentations affected the time of diagnosis once patients consulted health care providers. Patients presented with good functional status, no cough and had unusual symptoms obtained delayed diagnosis from the health system. These appearances may hinder PTB suspicion by heath care providers or they may fail to suspect PTB as of poor skill in identifying rare symptoms. Normal chest X-ray and smear negative results at initial diagnosis have also delayed PTB diagnosis. This may be related to the poor skill of healthcare providers in detecting the disease or poor sensitivity of diagnostic methods to detect cases at early stage [42].

Our review indicated that considerable number PTB patients in LMICs sought initial care from private clinics, drug vendors and lower level public health facilities where TB diagnosis services are unavailable [30]. So, if these health providers are engaged in TB services, they will contribute greatly in identification of presumptive cases and refer to nearby higher health facilities in their setting. Patients who sought care from multiple providers are likely to experience health system delay [35]. In addition, unemployment, labor migration and low income were also reported as factors of health system delay. This might be due to the high costs for pathological and x-ray diagnosis which are not free of charge globally unlike the rest of TB services [43].

Despite these findings and scientific arguments, it appears to have some methodological limitations that arose from either individual studies or the review process. Only English articles were reviewed. There could be many articles written in other languages that might be different with respect to size and quality. All included studies except one were cross-sectional. Therefore, methodological bias and confounding which are common in cross-sectional studies might have been introduced. Furthermore, there was lack of detailed information and inconsistent reporting among retrieved studies. Some studies computed separate median patient delay (stratified analysis), few reported *p*-value only [44], no clear cutoff point to categorize delay, and the variability of predictor variables across the studies. This limited the number of studies and predictor variables considered in the meta-analysis that could affect the power of the pooled analysis.

## Conclusion

This systematic review and meta-analysis confirms delays in diagnosis of PTB have remained higher similar to the preceding periods. Both patient and health system delays have contributed for observed delay all over the high burden low- and middle- income countries. This implies that the existing strategies and efforts under implementation did not appear to improve the desired early PTB case presentation, detection and prompt treatment initiation in low- and middle- income countries.

Multiple patient, provider and health system related factors seemed to contribute for the observed high diagnosis delays. The pooled analysis confirmed poor literacy and preference to seek care from informal healers significantly associated with patient delay. Substantial numbers of PTB patients still sought initial TB care from private healthcare facilities, traditional/religious healers and low level/community health workers. Hence, systematic engagement of these parties would potentially improve case finding strategies. If they are engaged in presumptive case identification and active referral network with TB care facilities established, early care seeking and detection can substantially be improved. Continuous community awareness activities should also be strengthened.

## Abbreviations

CI: Confidence interval
HIV: Human immuno-deficiency virus
IQR: Inter-Quartile Range
LMICS: Low- and Middle-Income Countries
NTP: National tuberculosis control programs
OR: Odds Ratio
PRISMA: Preferred reporting items for systematic reviews and meta-analyses
PTB: Pulmonary Tuberculosis
TB: Tuberculosis
WHO: World Health Organization
